# Comparison of the Effects of Dorzolamide/Timolol Fixed Combination versus Latanoprost on Intraocular Pressure and Ocular Perfusion Pressure in Patients with Normal-Tension Glaucoma: A Randomized, Crossover Clinical Trial

**DOI:** 10.1371/journal.pone.0146680

**Published:** 2016-01-12

**Authors:** Na Young Lee, Hae-Young Lopilly Park, Chan Kee Park

**Affiliations:** 1 Department of Ophthalmology and Visual Science, College of Medicine, Incheon St. Mary's Hospital, The Catholic University of Korea, Incheon, Korea; 2 Department of Ophthalmology and Visual Science, College of Medicine, Seoul St. Mary’s Hospital, The Catholic University of Korea, Seoul, Korea; Sun Yat-sen University, CHINA

## Abstract

**Backgroud:**

To assess the noninferiority of a dorzolamide-timolol fixed combination (DTFC) versus latanoprost in terms of intraocular pressure (IOP) and to compare blood pressure (BP), ocular perfusion pressure (OPP) and diastolic ocular perfusion pressure (DOPP) between the latanoprost and DTFC groups in patients with normal-tension glaucoma (NTG).

**Methods:**

Prospective, interventional, randomized, single-blinded, crossover design study. Patients with newly diagnosed NTG that had not been treated with a glaucoma medication in the most recent 2 months were recruited. In total, 44 patients with NTG were randomly allocated to one of two groups. Patients in group A were treated with DTFC, lubricant, and latanoprost for 4 weeks each, whereas patients in group B were treated with latanoprost, lubricant, and DTFC for 4 weeks each. Patients were examined on day 1 (without medication), week 4 (under medication), week 8 (without medication), and week 12 (under medication). At weeks 4 and 12, diurnal IOP, systolic and diastolic BP, and OPP were measured at 8:00 AM, 10:00 AM, 12:00 PM, 4:00 PM, and 8:00 PM.

**Results:**

Baseline demographic characteristics showed no difference in terms of age, sex, central corneal thickness, spherical equivalent, or stage of glaucoma between the groups. The between-group difference was -0.19 ± 0.18 mmHg (mean ± SE, upper bound of one-sided 95% CI, 0.12). Diurnal IOP showed no difference between the groups with an average IOP reduction of 13.1% using latanoprost and 12.3% using DTFC. Diurnal systolic and diastolic BP were lower in the DTFC group than the latanoprost group; however, the difference between the groups was not statistically significant. Diurnal OPP and DOPP also showed no statistically significant difference between the groups.

**Conclusions:**

IOP lowering efficacy of DTFC was noninferior to that of latanoprost in newly diagnosed NTG patients. There was no difference in BP, OPP, or DOPP between the latanoprost and DTFC groups. This prospective, randomized, single-blinded, crossover study demonstrated the noninferiority of DTFC versus latanoprost in terms of IOP in patients with NTG.

**Trial Registration:**

ClinicalTrials.gov NCT01175902

## Introduction

Glaucoma, which causes optic nerve damage and visual field loss, is one of the main causes of blindness and irreversible deterioration in vision worldwide [1]. Normal-tension glaucoma (NTG) is a clinical entity characterized by glaucomatous optic nerve damage and visual field defects with an intraocular pressure (IOP) in the statistically normal range; NTG accounts for 77% of cases of primary open-angle glaucoma (POAG) in Korean patients [2]. Many researchers have investigated IOP-independent factors in patients with NTG; however, the only proven treatment that can effectively prevent the development and progression of glaucoma remains a reduction in IOP [3,4].

Since 1996, latanoprost has been approved for clinical use in the United States and Europe and was introduced as the first prostaglandin analog in Korea. In Japan, prostaglandin analogs have become the first-line treatment for NTG because of their ability to reduce IOP [5]. As in Japan, a survey of the Korean Glaucoma Society resulted in agreement that the first-line treatment for NTG is a prostaglandin analog, especially latanoprost. Additionally, a meta-analysis of medical interventions for NTG showed that prostaglandin analogs were the most effective medications for lowering IOP, with mean relative reduction at both peak and trough of approximately 20% [6].

Dorzolamide/timolol fixed combination (DTFC) has been established as an effective IOP-lowering agent in patients with POAG with high IOP [7,8]. Moreover, DTFC is reportedly an effective IOP-lowering agent in patients with NTG [9]. However, there is little published information regarding the efficacy of DTFC and no reports of a comparison with latanoprost in patients with NTG.

In addition to comparison of IOP, vascular instability has been suggested as one of the characteristics of NTG; thus, the simultaneous evaluation of ocular perfusion pressure (OPP) is also valuable [10–12]. The purpose of the present study was to assess the noninferiority of the dorzolamide 2%/timolol maleate 0.5% fixed combination (Cosopt; Merck & Co., Inc., Blue Bell, PA) versus latanoprost 0.005% (Xalatan; Pfizer, Inc., New York, NY) in terms of IOP and to compare blood pressure (BP), OPP and diastolic OPP (DOPP) between the DTFC and latanoprost as an initial treatment in patients with NTG.

## Materials and Methods

### Patients

We conducted a single-center, prospective, interventional, randomized, single-blinded crossover study. This clinical trial was registered at ClinicalTrials.gov (“Results Record cosopt-IOP/OPP,” NCT01175902). The Institutional Review Board of Seoul St. Mary’s Hospital approved this study, which adhered to the principles of the Declaration of Helsinki. All patients signed an institutional review board-approved informed consent agreement form before any procedure was performed.

Patients with NTG were recruited from the glaucoma clinic of Seoul St. Mary’s Hospital between April 2011 and October 2014. The identification of NTG was based on reproducible glaucomatous visual field defects corresponding to typical optic nerve head changes. The presence of unilateral or bilateral visual field loss (described below) was determined by at least two consecutive automatic perimetry values using the Swedish Interactive Threshold Algorithm Standard 24–2 visual field test on a Humphrey Field Analyzer (Carl Zeiss Meditec, Dublin, CA). One eye was selected randomly in cases where both eyes were treated.

The inclusion criteria were (1) an age of 45 to 75 years, (2) best-corrected visual acuity no worse than 20/30 Snellen equivalent, (3) optic nerve head cupping (i.e., a vertical cup-to-disc ratio of >0.6) and/or notching of the neuroretinal rim and/or retinal nerve fiber defects characteristic of glaucoma, (4) visual field loss (i.e., a glaucoma hemifield test result outside normal limits, a pattern standard deviation probability of <5%, or a cluster of three or more non-edge points in a location typical of glaucoma, all of which were depressed on the pattern deviation plot at a P level of <5% and at least one of which was depressed at a P level of <1% on two consecutive visual field tests), (5) repeated measurements of untreated IOP with documented values of <22 mmHg, (6) central corneal thickness ranging from 540 to 560 μm, and (7) open angle confirmed by gonioscopy.

The exclusion criteria were (1) active or chronic systemic diseases and/or currently taking medication known to affect IOP, BP, and/or heart rate (HR); (2) corneal abnormalities preventing reliable applanation tonometry; (3) a history of severe ocular trauma, ocular inflammation or infection, intraocular surgery, argon laser treatment, or laser trabeculoplasty; (4) myopic or other fundus changes preventing reliable optic disc evaluation; (5) visual field defects caused by nonglaucomatous disease; and (6) a history of allergy to the ingredients of DTFC or latanoprost eye drops.

### Procedures

**Fig 1:**
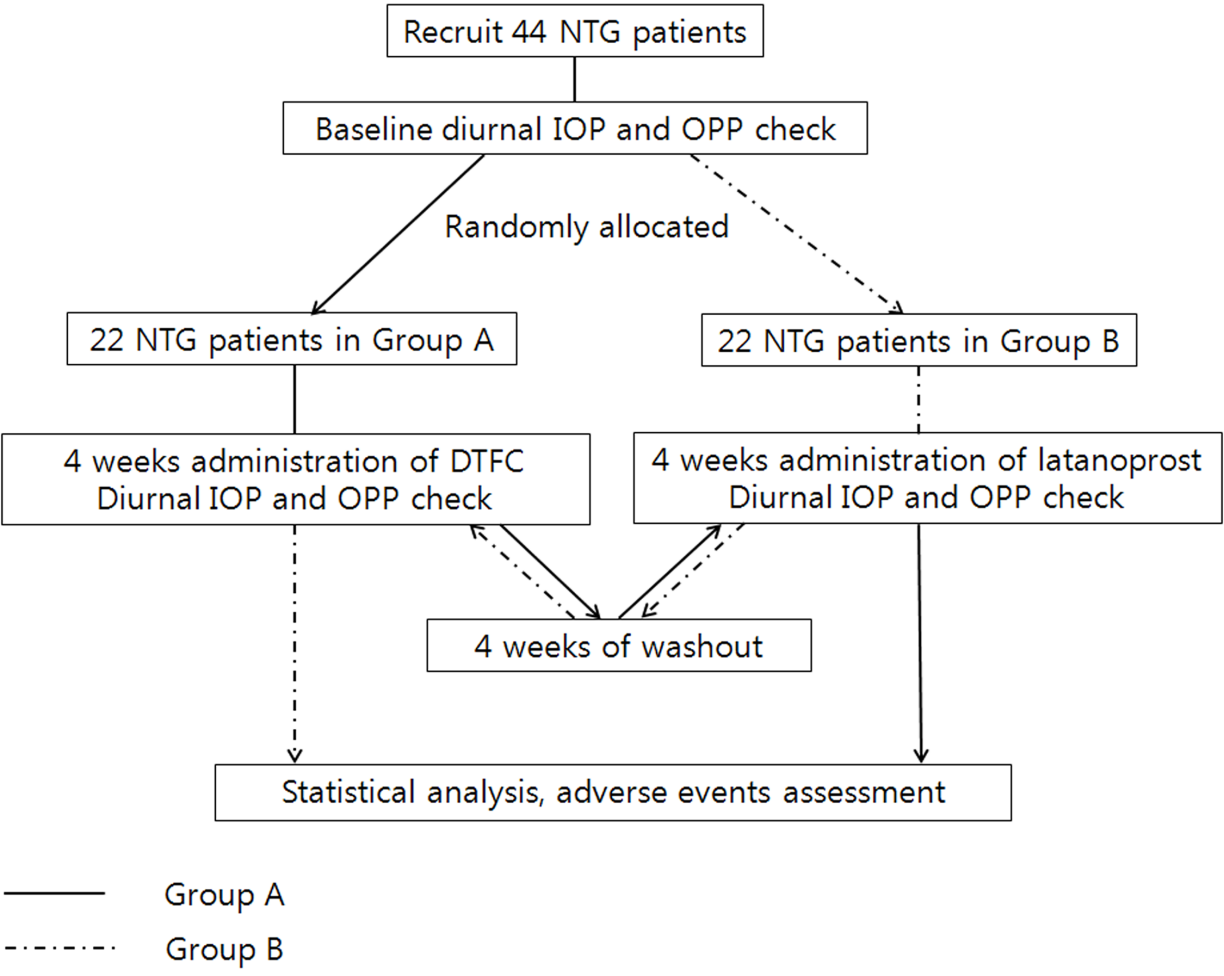
Consort diagram. NTG indicates normal tension glaucoma; IOP, intraocular pressure; OPP, ocular perfusion pressure; DTFC, dorzolamide/timolol fixed combination.

We recruited patients with newly diagnosed NTG that had not been treated with a glaucoma medication in the past 2 months, then enrolled patients according to the above-listed inclusion and exclusion criteria (Fig 1). All patients underwent a full ophthalmic examination, including visual acuity, refraction, keratometry, detailed stereoscopic biomicroscopy of the anterior segment, IOP measurements with Goldmann applanation tonometry, dilated fundus examination, fundus photography, stereoscopic photography of the optic disc (VK-2; Kowa Optimed, Inc., Torrance, CA), and perimetry using the Swedish Interactive Threshold Algorithm Standard 24–2 test on a Humphrey Field Analyzer.

On day 1, all participants were checked for baseline diurnal IOP, systolic and diastolic BP (at 8:00 AM, 12:00 PM, and 4:00 PM) and then randomized using computer-generated random assignment to one of the treatment regimens: DTFC (at 8:00 AM and 8:00 PM) and latanoprost 0.005% (at 10:00 PM). IOP was measured by Goldmann applanation tonometry (mean of three consecutive readings) with the patient in a sitting position at the slit lamp. Every IOP was measured by one blinded glaucoma specialist who was unaware of the treatment assignments. After the IOP measurements and a 5-min rest, the pulse rate and systolic and diastolic BP of the radial artery were measured in the sitting position using a standard automated blood pressure cuff. During the study, all measuring instruments were kept calibrated in accordance with the manufacturer’s instructions.

OPP and DOPP were calculated using the following formulae [12,13]:

OPP=(13systolicBP+23diastolicBP)23−IOP

DOPP=diastolicBP−IOP

After 4 weeks of treatment with DTFC or latanoprost, all participants were checked for diurnal IOP, systolic and diastolic BP, and HR (at 8:00 AM, 10:00 AM, 12:00 PM, 4:00 PM, and 8:00 PM) in week 4. During the 4-week washout period, the patients used only lubricants. At the end of the washout period, all participants were checked for baseline diurnal IOP and systolic and diastolic BP (at 8:00 AM, 12:00 PM, and 4:00 PM; week 8). After another 4 weeks of treatment with the alternative medication, all participants were checked again for diurnal IOP, systolic and diastolic BP, and HR (at 8:00 AM, 10:00 AM, 12:00 PM, 4:00 PM, and 8:00 PM; week 12). Four diurnal assessments of IOP, BP, and HR were thus obtained for each patient: at baseline, at washout, and at the end of each treatment period. Fluctuation in IOP was calculated as the “highest daily IOP–lowest daily IOP” for each visit. Complete ocular and systemic examinations were performed at baseline and at the end of each phase of the trial, and any ocular or systemic events were noted.

### Statistics

A noninferiority test tests that the treatment mean is not worse than the reference mean by more than a small equivalence margin. We used two crossover-sample means which compute sample size for noninferiority test in 2×2 cross-over designs. To demonstrate the noninferiority of the DTFC to the latanoprost, the sample size calculation was based on the assumption of a noninferiority margin of a trough IOP of 1.5 mmHg. With a sample size of 21 patients per group, this study had 80% power (1 –β = 0.80) and an α = 0.05 crossover-design analysis [12,14–16]. Baseline characteristics were compared using an independent t-test and Chi-square test. Noninferiority of IOP was proved by calculating the one-sided 95% CI for the two treatments. In this study, the upper limit of the 95% CI was expected to be below the maximal acceptable clinically significant difference of 1.5 mmHg for IOP. For all analyses, *P* value of < 0.05 was considered statistically significant.

Safety analyses included all patients receiving at least one dose of the study medication (safety population). The Medical Dictionary for Regulatory Activities coding system was used to classify adverse events, and frequencies of ocular and systemic adverse events were tabulated.

## Results

### Patients

In total, 44 patients with NTG were recruited in this study. Their demographic data are summarized in Table 1. Baseline demographic characteristics showed no difference in terms of age, sex, central corneal thickness, spherical equivalents, or stage of glaucoma between the groups. All patients completed the two crossover phases, and no important adverse events were observed.

**Table 1:** Baseline demographics and clinical characteristics of the study population. VF = visual field; RNFL = retinal nerve fiber layer.

	Latanoprost	Dorzolamide/Timolol	*P* Value
	→ Dorzolamide/Timolol	→ Latanoprost	
Age	60.72 ± 7.42	59.68 ± 7.12	0.636°
Gender, Male:Female	7:15	4:18	0.244^†^
Spherical equivalent	-0.74 ± 1.95	-1.04 ± 2.71	0.676°
Central corneal thickness	542.73 ± 11.43	544.23 ± 17.33	0.737°
Mean deviation of VF	-4.00 ± 3.79	-2.92 ± 2.96	0.300°
Pattern standard deviation of VF	4.86 ± 4.04	3.72 ± 3.28	0.313°
Average RNFL thickness	79.14 ± 10.33	82.77 ± 11.20	0.276°

°Independent t-test.

^†^Chi-square test.

### IOP

**Fig 2:**
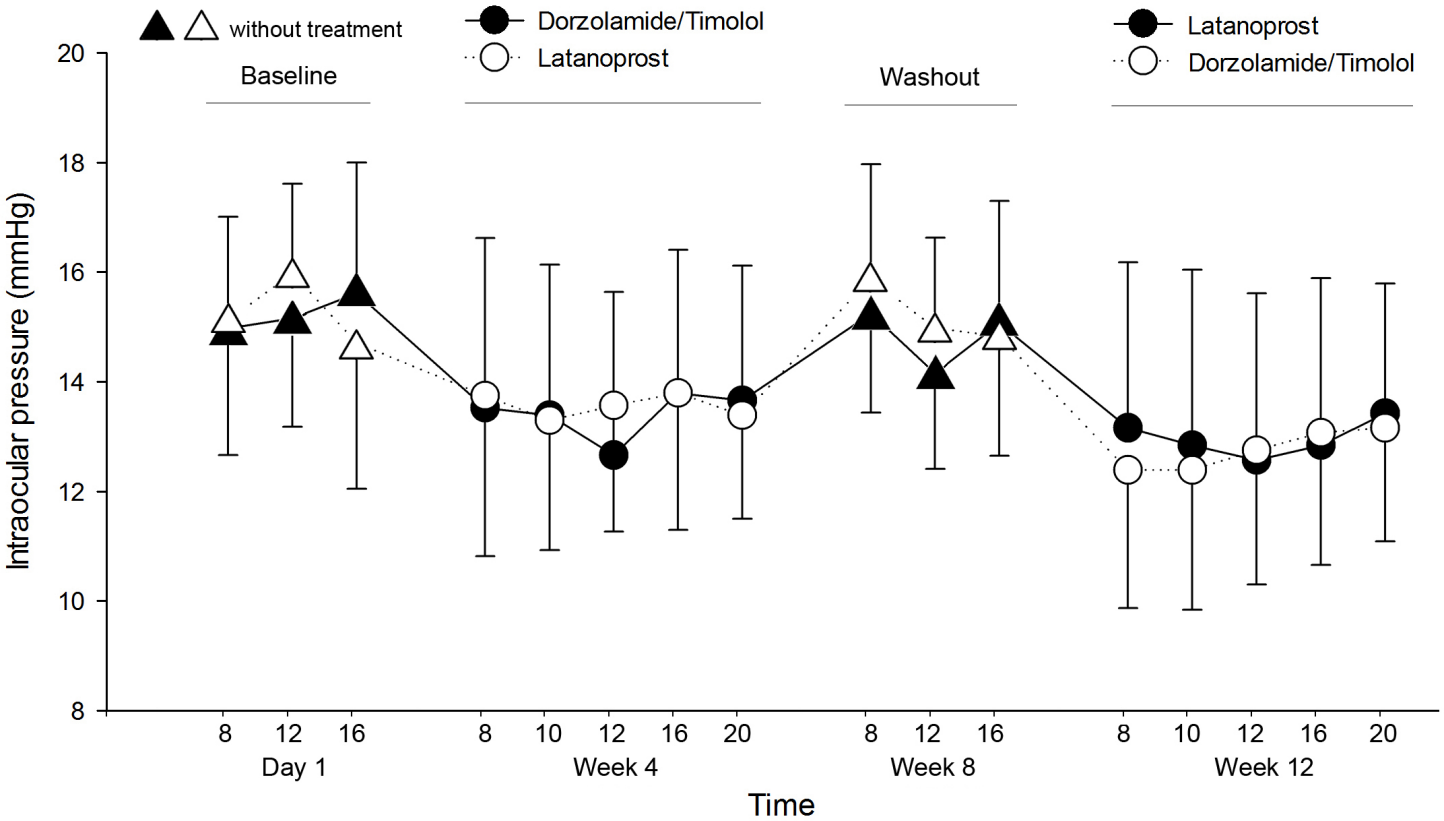
Mean intraocular pressure during the circadian curve for the three crossover phases: at baseline, after dorzolamide/timolol, and after latanoprost. Vertical bars indicate the 95% confidence interval of the estimates.

The histogram shown in Fig 2 describes the diurnal IOP in each group during 12 weeks. The mean IOP at all time points was lower than at baseline. We compared the baseline and washout IOP to evaluate potential carry-over effect using paired t-test. The difference of IOP between the two treatments was 0.39 ± 0.21 mmHg (mean ± SE) and there was no statistically significant difference (p = 0.07). Diurnal IOP showed no difference between the groups at all time points, with an average IOP reduction of 13.1% using latanoprost and 12.3% using DTFC (Table 2). The difference of IOP between the two treatments was -0.19 ± 0.18 mmHg (mean ± SE, upper bound of one-sided 95% CI, 0.12). In addition, DTFC met the criteria for noninferiority to latanoprost at each time points (Table 2). Because the upper limit of this 95% CI was ≤ 1.5 mmHg, the IOP-lowering efficacy of DTFC was proved to be noninferior to that of latanoprost. Fluctuation in IOP was calculated for each visit, and there was no statistically significant difference between the two treatments (p = 0.65).

**Table 2:** Intraocular pressure diurnal profiles of two groups. DTFC = dorzolamide-timolol fixed combination

	Latanoprost	Dorzolamide/Timolol	*P* Value°
	→ Dorzolamide/Timolol	→ Latanoprost	
**At baseline**			
8am	15.50 ± 3.30	15.27 ± 2.88	0.809
12pm	15.95 ± 2.80	15.13 ± 2.45	0.309
16pm	14.68 ± 2.66	15.59 ± 2.38	0.239
**After 4 weeks**			
**with first medication**			
8am	13.72 ± 2.93	13.50 ± 3.09	0.804
10am	13.27 ± 2.37	13.36 ± 2.75	0.907
12pm	13.54 ± 2.30	12.63 ± 2.98	0.265
16pm	13.77 ± 2.50	13.77 ± 2.61	0.999
20pm	13.36 ± 1.89	13.63 ± 2.46	0.682
**After 8 weeks with wash-out**			
8am	15.86 ± 2.45	15.22 ± 2.72	0.420
12pm	14.95 ± 2.57	14.13 ± 2.47	0.348
16pm	14.40 ± 2.46	15.18 ± 2.61	0.318
**After 12 weeks**			
**with second medication**			
8am	12.36 ± 2.52	13.13 ± 3.02	0.363
10am	12.36 ± 2.55	12.81 ± 3.21	0.607
12pm	12.72 ± 2.45	12.54 ± 3.05	0.829
16pm	13.04 ± 2.41	12.81 ± 3.05	0.794
20pm	13.13 ± 2.07	13.40 ± 2.36	0.686
***P* Value**^**†**^	0.499	0.057	
***P* Value**^**‡**^		0.266	
**Differences of**		**Upper one-sided 95% CI**	**Non-Inferiority**
**DTFC and latanoprost**			**(margin = 1.5)**
8am	0.50 ± 2.94	1.25	proved
10am	0.18 ± 2.64	0.85	proved
12pm	0.36 ± 2.73	1.06	proved
16pm	-0.11 ± 2.76	0.58	proved
20pm	0.00 ± 2.53	0.64	proved

°Independent t-test.

^†^Paired t-test for the difference between Latanoprost and Dorzolamide/Timolol at 8 am within the same group.

^**‡**^Paired t-test for the difference between Latanoprost and Dorzolamide/Timolol at 8 am in the two groups combined.

### BP, HR, and OPP

**Fig 3:**
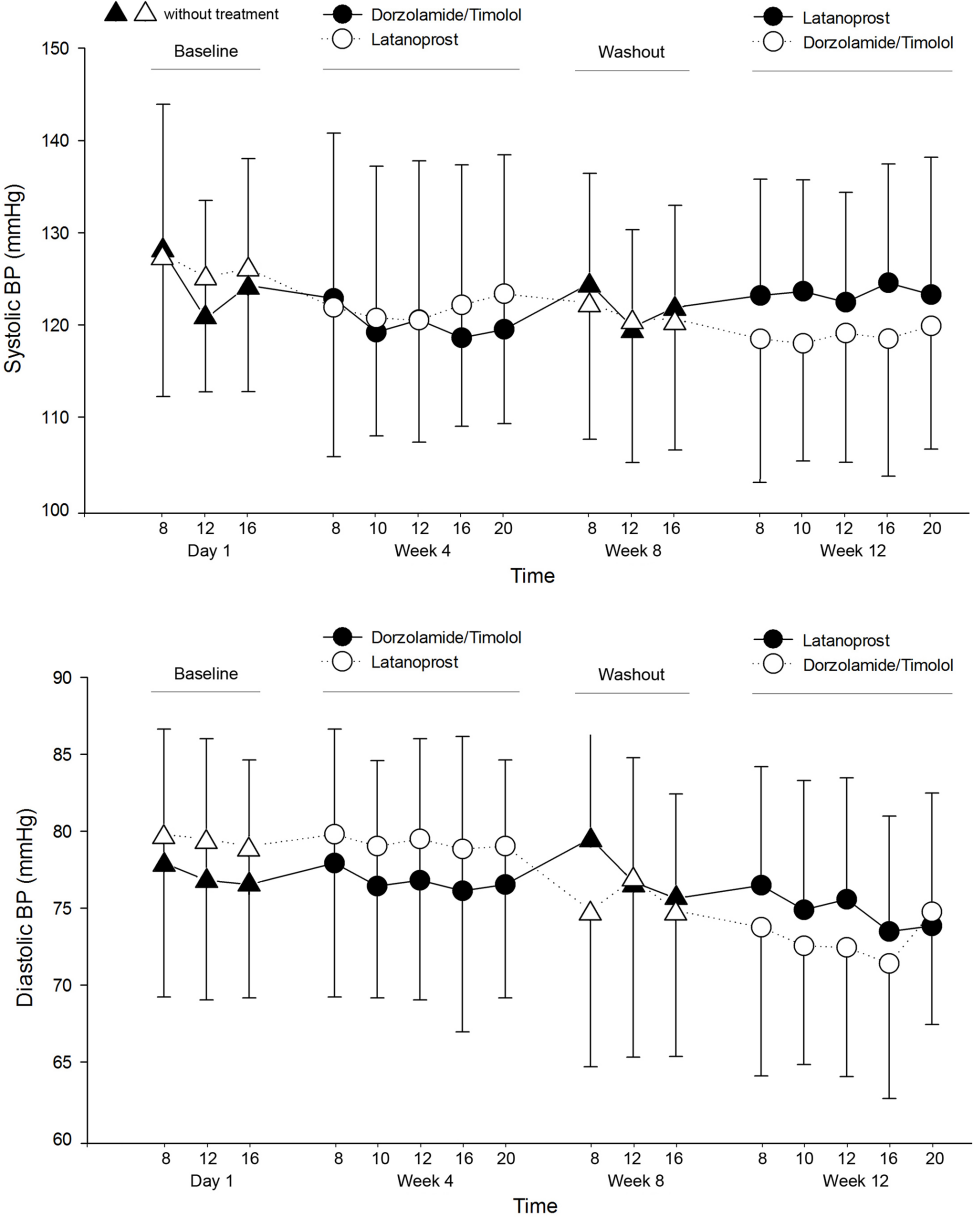
Mean systolic and diastolic blood pressure (BP) during the circadian curve for the three crossover phases: at baseline, after dorzolamide/timolol, and after latanoprost. Vertical bars indicate the 95% confidence interval of the estimates.

**Fig 4:**
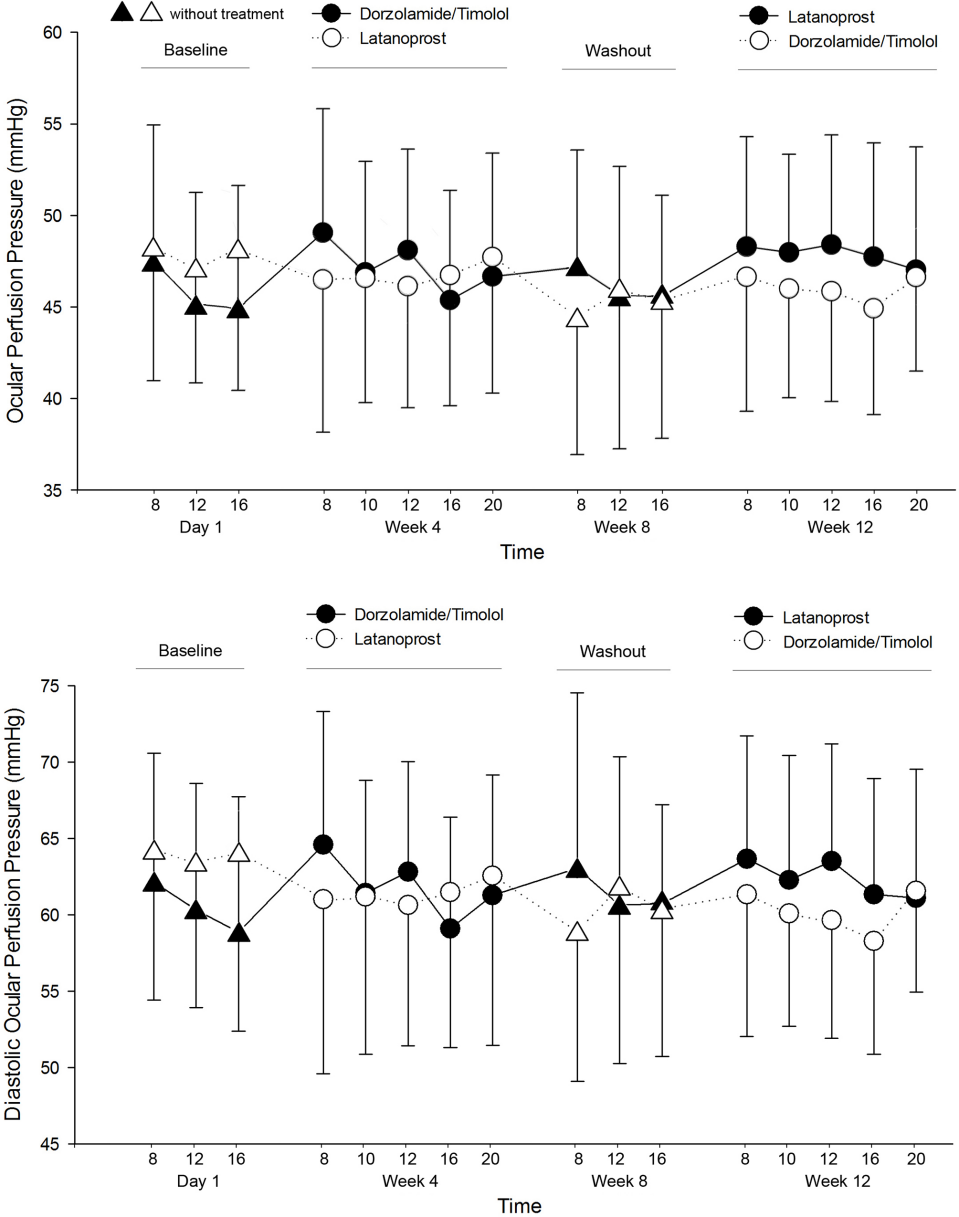
Mean ocular perfusion pressure and diastolic ocular perfusion pressure during the circadian curve for the three crossover phases: at baseline, after dorzolamide/timolol, and after latanoprost. Vertical bars indicate the 95% confidence interval of the estimates.

Diurnal systolic and diastolic BP were lower in the DTFC than latanoprost group; however, the difference between the groups was not statistically significant (Table 3, Fig 3). The HR showed no statistically significant difference between the groups (Table 4). In terms of OPP and DOPP, mean OPP was 46.68 ± 6.15 mmHg in DTFC group, and 47.39 ± 6.61 mmHg in latanoprost group. There was no statistically significant difference between the groups (p = 0.248). Mean DOPP was 61.05 ± 7.78 mmHg in DTFC group, and 61.91 ± 9.19 mmHg in latanoprost group. There was no statistically significant difference between the groups (p = 0.290). Diurnal OPP and DOPP also showed no statistically significant difference between the groups at all time points (Tables 5 and 6; Fig 4).

**Table 3:** Systolic BP and diastolic BP diurnal profiles of two groups.

	Latanoprost	Dorzolamide/Timolol	*P* Value°
	→ Dorzolamide/Timolol	→ Latanoprost	
**At baseline**			
8am	127.64 ± 15.45 / 79.77 ± 10.59	127.95 ± 15.89 / 77.90 ± 8.70	0.947 / 0.401
12pm	125.14 ± 12.44 / 79.45 ± 10.45	120.64 ± 12.78 / 76.77 ± 9.20	0.243 / 0.692
16pm	125.36 ± 14.65 / 78.81 ± 11.89	123.68 ± 15.63 / 76.09 ± 10.04	0.715 / 0.362
**After 4 weeks**			
**with first medication**			
8am	121.82 ± 16.15 / 79.77 ± 10.59	122.82 ± 17.9 / 77.90 ± 8.70	0.847 / 0.401
10am	120.68 ± 12.76 / 79.00 ± 9.88	119.14 ± 18.01 / 76.40 ± 8.15	0.745 / 0.812
12pm	120.45 ± 13.22 / 79.45 ± 10.45	120.50 ± 17.23 / 76.77 ± 9.20	0.984 / 0.692
16pm	122.09 ± 13.14 / 78.81 ± 11.89	118.55 ± 18.74 / 76.09 ± 10.04	0.472 / 0.362
20pm	123.32 ± 14.09 / 79.00 ± 9.88	119.45 ± 18.92 / 76.50 ± 8.09	0.447 / 0.546
**After 8 weeks with wash-out**			
8am	122.32 ± 14.76 / 74.81 ± 10.16	124.45 ± 11.92 / 79.50 ± 7.71	0.601 / 0.093
12pm	120.50 ± 15.47 / 76.86 ± 11.59	119.68 ± 10.57 / 76.68 ± 8.07	0.839 / 0.952
16pm	119.86 ± 14.56 / 74.81 ± 9.49	121.82 ± 13.08 / 75.59 ± 6.80	0.642 / 0.758
**After 12 weeks**			
**with second medication**			
8am	118.41 ± 15.47 / 73.72 ± 9.67	123.14 ± 12.58 / 76.45 ± 7.71	0.273 / 0.307
10am	117.95 ± 12.73 / 72.50 ± 7.70	123.59 ± 12.06 / 74.86 ± 8.40	0.139 / 0.336
12pm	119.05 ± 13.99 / 72.40 ± 8.39	122.41 ± 11.89 / 75.54 ± 7.89	0.395 / 0.209
16pm	118.45 ± 14.90 / 71.36 ± 8.78	124.50 ± 12.89 / 73.45 ± 7.50	0.156 / 0.401
20pm	119.82 ± 13.34 / 74.72 ± 7.32	123.23 ± 14.88 / 73.81 ± 8.64	0.428 / 0.709
***P* Value**^**†**^	0.547 / 0.556	0.200 / 0.482	
***P* Value**^**‡**^		0.379 / 0.949	

°Independent t-test.

^†^Paired t-test for the difference between Latanoprost and Dorzolamide/Timolol at 8 am within the same group.

^**‡**^Paired t-test for the difference between Latanoprost and Dorzolamide/Timolol at 8 am in the two groups combined.

**Table 4:** Heart rate diurnal profiles of two groups.

	Latanoprost	Dorzolamide/Timolol	*P* Value°
	→ Dorzolamide/Timolol	→ Latanoprost	
**At baseline**			
8am	72.22 ± 10.97	72.77 ± 8.19	0.853
12pm	70.27 ± 9.77	69.54 ± 7.78	0.786
16pm	72.45 ± 12.62	71.77 ± 9.72	0.842
**After 4 weeks**			
**with first medication**			
8am	72.04 ± 10.20	72.27 ± 8.24	0.936
10am	72.54 ± 11.97	67.59 ± 8.85	0.126
12pm	71.09 ± 10.32	69.22 ± 9.58	0.538
16pm	71.36 ± 9.01	68.50 ± 9.75	0.318
20pm	71.59 ± 9.50	69.81 ± 9.16	0.532
**After 8 weeks with wash-out**			
8am	71.09 ± 11.07	73.90 ± 11.08	0.404
12pm	68.95 ± 6.55	68.45 ± 9.92	0.845
16pm	70.63 ± 7.49	70.77± 9.60	0.958
**After 12 weeks**			
**with second medication**			
8am	71.77 ± 6.74	71.86 ± 6.31	0.963
10am	70.81± 6.22	70.77 ± 7.31	0.982
12pm	68.77 ± 4.99	71.36 ± 8.78	0.236
16pm	71.13 ± 7.27	70.72 ± 7.36	0.854
20pm	69.77 ± 7.85	69.50 ± 7.58	0.907
***P* Value**^**†**^	0.822	0.885	
***P* Value**^**‡**^		0.958	

°Independent t-test.

^†^Paired t-test for the difference between Latanoprost and Dorzolamide/Timolol at 8 am within the same group.

^**‡**^Paired t-test for the difference between Latanoprost and Dorzolamide/Timolol at 8 am in the two groups combined.

**Table 5:** Ocular perfusion pressure diurnal profiles of two groups.

	Latanoprost	Dorzolamide/Timolol	*P* Value°
	→ Dorzolamide/Timolol	→ Latanoprost	
**At baseline**			
8am	48.31 ± 7.27	47.58 ± 7.42	0.149
12pm	47.16 ± 6.24	45.22 ± 6.10	0.454
16pm	48.21 ± 7.70	45.00 ± 6.70	0.085
**After 4 weeks**			
**with first medication**			
8am	46.57 ± 8.34	49.12 ± 6.76	0.272
10am	46.65 ± 6.81	46.95 ± 6.06	0.877
12pm	46.21 ± 6.64	48.17 ± 5.51	0.294
16pm	46.81 ± 7.14	45.44 ± 5.98	0.495
20pm	47.79 ± 7.43	46.73 ± 6.73	0.619
**After 8 weeks with wash-out**			
8am	44.56 ± 7.55	47.23 ± 6.40	0.214
12pm	45.98 ± 8.66	45.71 ± 7.02	0.909
16pm	45.47 ± 7.57	45.64 ± 5.52	0.934
**After 12 weeks**			
**with second medication**			
8am	46.71 ± 7.34	48.36 ± 6.00	0.419
10am	46.07 ± 5.96	48.04 ± 5.36	0.256
12pm	45.91 ± 6.00	48.47 ± 5.98	0.163
16pm	44.99 ± 5.80	47.81 ± 6.21	0.127
20pm	46.70 ± 5.14	47.10 ± 6.70	0.824
***P* Value**^**†**^	0.435	0.904	
***P* Value**^**‡**^		0.547	

°Independent t-test.

^†^Paired t-test for the difference between Latanoprost and Dorzolamide/Timolol at 8 am within the same group.

^**‡**^Paired t-test for the difference between Latanoprost and Dorzolamide/Timolol at 8 am in the two groups combined.

**Table 6:** Diastolic ocular perfusion pressure diurnal profiles of two groups.

	Latanoprost	Dorzolamide/Timolol	*P* Value°
	→ Dorzolamide/Timolol	→ Latanoprost	
**At baseline**			
8am	64.27 ± 9.84	62.18 ± 8.43	0.454
12pm	63.50 ± 9.54	60.36 ± 8.28	0.251
16pm	64.13 ± 11.71	58.90 ± 8.86	0.103
**After 4 weeks**			
**with first medication**			
8am	61.04 ± 11.41	64.63 ± 8.73	0.248
10am	61.22 ± 10.32	61.45 ± 7.41	0.934
12pm	60.68 ± 9.24	62.86 ± 7.21	0.388
16pm	61.50 ± 10.17	59.13 ± 7.29	0.382
20pm	62.59 ± 11.12	61.31 ± 7.87	0.664
**After 8 weeks with wash-out**			
8am	58.95 ± 9.84	63.09 ± 8.89	0.151
12pm	61.90 ± 11.61	60.68 ± 9.70	0.706
16pm	60.40 ± 9.66	60.77 ± 6.48	0.884
**After 12 weeks**			
**with second medication**			
8am	61.36 ± 9.30	63.48 ± 8.07	0.383
10am	60.13 ± 7.41	62.31 ± 8.15	0.358
12pm	59.68 ± 7.72	63.54 ± 7.69	0.104
16pm	58.31 ± 7.42	61.36 ± 7.59	0.186
20pm	61.59 ± 6.63	61.13 ± 8.45	0.844
***P* Value**^**†**^	0.492	0.829	
***P* Value**^**‡**^		0.522	

°Independent t-test.

^†^Paired t-test for the difference between Latanoprost and Dorzolamide/Timolol at 8 am within the same group.

^**‡**^Paired t-test for the difference between Latanoprost and Dorzolamide/Timolol at 8 am in the two groups combined.

### Adverse Events

Of the 44 patients included, 21 (47%) reported adverse events. Eye irritation was the most frequently reported adverse event, followed by ocular hyperemia and the majority of eye irritations were mild in intensity. No serious adverse events occurred in this study and no systemic adverse event was considered to be related to the study medication. No patient discontinued DTFC or latanoprost due to an adverse event (Table 7).

**Table 7:** Number (%) of Patients with Ocular Treatment-Related Adverse Events.

	Latanoprost	Dorzolamide/Timolol	*P* Value°
**Eye irritation**	6 (27.3%)	10 (45.5%)	0.303
Mild	6 (27.3%)	8 (36.4%)	
Moderate	0 (0%)	2 (9.1%)	
Severe	0 (0%)	0 (0%)	
**Ocular hyperemia**	4 (18.1%)	1 (4.5%)	0.345
Mild	3 (13.6%)	1 (4.5%)	
Moderate	1 (4.5%)	0 (0%)	
Severe	0 (0%)	0 (0%)	

°Fisher’s exact test.

## Discussion

The present randomized study found no statistically significant difference in IOP or OPP between the DTFC and latanoprost group and noninferiority of DTFC to latanoprost in IOP lowering efficacy in NTG patients. To our knowledge, this is the first report on the efficacy and OPP of DTFC used as a first-line medication in patients with NTG.

DTFC has generally been shown to be an efficacious and well-tolerated hypotensive medication. Consequently, combination therapy is prescribed as the first-line therapy in the practices of many ophthalmologists under real-life private practice conditions [17]. Henderer et al.[18] reported that DTFC was effective in rapidly reducing IOP by approximately 40% after 2 months of treatment and maintaining low pressure when used as the first-line therapy in patients with glaucoma with an IOP of >30 mmHg.

The purpose of our study was to compare the effects of DTFC versus latanoprost on IOP and OPP in patients with NTG. We found that both DTFC and latanoprost had strong, statistically significant IOP-lowering effects. However, few studies have reported the efficacy of DTFC in patients with NTG, and there is no report on the effects of DTFC in patients with NTG in terms of OPP [9]. The IOP-lowering effect of DTFC in patients with NTG is known to be inferior to that in previous studies of patients with POAG. The present study also showed less IOP reduction with DTFC compared with previous studies that included patients with POAG; however, there was a noninferior reduction versus latanoprost. Additionally, substantial IOP peaks or larger diurnal fluctuations may increase the rate of glaucoma progression [19]. We calculated fluctuation in IOP for each visit and confirmed that there was no statistically significant difference between the two treatments. In POAG, DTFC is regarded as having the same or superior IOP reduction efficacy as latanoprost [8]. However, in patients with NTG, prostaglandin analogs are the first-line treatment regimen in Korea and Japan. The main concern in using DTFC in patients with NTG is the systemic effects of timolol. The topical administration of timolol can cause cardiovascular and hemodynamic changes, and several side effects have been reported [20–22].

A reduction in IOP does not stop glaucomatous disease progression in many patients with glaucoma. Thus, the role of ocular blood flow in glaucoma has recently been discussed as a potential risk factor for the development and continued progression of the disease in certain patients, especially those with NTG [23]. Some patients, despite a significant reduction in IOP, show progression of anatomical and functional damage, suggesting that factors other than IOP are involved in this disease. For these reasons, DOPP may be more important than IOP alone in determining optic nerve head damage [13].

When compared with timolol in POAG, DTFC augments ocular tension reduction and reduces the amount of time required for blood to pass through the superior retinal vasculature [24]. Costagliola et al.[12] reported that timolol decreased the BP and HR with respect to baseline and that latanoprost increased the mean OPP, whereas timolol did not improve the OPP in Caucasians with NTG. In contrast, our results showed no significant decrease in the BP or HR with DTFC and no significant difference in OPP after DTFC or latanoprost. These findings could be explained by the combination of dorzolamide and timolol in DTFC [13]. Harris et al.[25] reported that dorzolamide accelerated inferotemporal retinal dye transit. Dorzolamide + timolol increased OPP compared with brimonidine + timolol in patients with POAG, and this might have resulted from the dorzolamide [26]. Dorzolamide increased the mean 24-h diastolic OPP level in patients with POAG, has been shown to increase ocular blood flow parameters, and is presumed to increase ocular blood flow through metabolic acidosis via elevated carbon dioxide levels in the eye tissues in patients with NTG [13,27]. Dorzolamide, unlike latanoprost, significantly reduced the arteriovenous passage times in the superior temporal retina in patients with NTG. Neither dorzolamide nor latanoprost had any statistically significantly effect on HR or BP [28]. DTFC and latanoprost had similar effects in terms of IOP reduction in patients with POAG; however, DTFC increased the pulse volume significantly, while latanoprost had no effect [16].

In the present study, the diurnal BP, OPP, and DOPP showed no statistical differences between the DTFC and latanoprost groups. Dorzolamide can compensate for the cardiovascular effects of timolol, and DTFC could be used in patients with NTG without concern about the effects of timolol.

Ikeda et al.[29] demonstrated that the incidence of latanoprost nonresponders among Japanese patients with OAG was higher than that among Caucasians. With similar patterns in Korea and Japan and a high incidence of NTG among patients with OAG, the limitation of treatment options for NTG to prostaglandin analogs, and mostly latanoprost, seems appropriate. Therefore, our results could provide theoretical support for the use of DTFC in patients with NTG. Latanoprost has the benefit once-daily use, whereas DTFC is administered twice daily. Although no important adverse events were observed with latanoprost or DTFC in this study, latanoprost and DTFC have different side effects. Thus, in patients with NTG, a hypotensive medication could be chosen among latanoprost and DTFC with tolerable side effects without concern about the IOP reduction and OPP.

The present study has several limitations. First, we did not measure the IOP or BP during 24-hour and did not obtain circardian IOP at the baseline and treatment periods. However, we checked the IOP and BP five times per day from 8:00 AM to 8:00 PM; this covered more measurement times during the day than in a previous study [9]. Second, the study population consisted entirely of Korean patients, which could potentially restrict the external validity of our study. However, despite these limitations, this study is informative regarding the management of NTG and may be helpful in future investigations of hypotensive medications.

In this study, we confirmed the noninferiority of DTFC versus latanoprost in terms of the IOP and there was no difference in terms of OPP between the two treatments. Additionally, DTFC showed as few adverse events as latanoprost. With no systemic contraindications, DTFC may be chosen as a first-line treatment in patients with NTG without concern about OPP efficacy.

## Supporting Information

S1 Text Study protocol-English. (DOC)

S2 Text Study protocol-original language-Korean. (DOC)

S3 Text CONSORT checklist. (DOC)
