# Supplementary material for: Comparison of the Effects of Dorzolamide/Timolol Fixed Combination versus Latanoprost on Intraocular Pressure and Ocular Perfusion Pressure in Patients with Normal-Tension Glaucoma: A Randomized, Crossover Clinical Trial
Source: PLoS One. 2016 Jan 12;11(1):e0146680. doi: 10.1371/journal.pone.0146680 (PMC4710520; doi:10.1371/journal.pone.0146680)
Supplement: S1 Text — (DOC) [file pone.0146680.s001.doc]

| **Section #1 - IISP Protocol Identification** | |
| --- | --- |
| **Study Title:** | A prospective, randomized, single masked, crossover design study to prove the non-inferiority of Cosopt compared to Xalatan in the aspects of intraocular pressure (IOP) and ocular perfusion pressure (OPP) in subjects with normal tension glaucoma (NTG) |
| **Request Date:** | **17/Dec/2009** |
| **Institution Name** | Department of ophthalmology and visual science, College of medicine.  The catholic university of Korea. |
| **Investigator Contact Information:**   - Full address - Phone No. - Fax No. - e-mail address | -505, banpo-dong, seocho-gu, department of ophthalmology,  Seoul st. Mary’s hospital, Seoul, Korea.  -82-2-2258-6199  -82-2-599-7405  [-ckpark@catholic.ac.kr](mailto:-ckpark@catholic.ac.kr) |

| **Section #2- Core Protocol** | |
| --- | --- |
| **2.1 Objectives & Hypotheses** | 2.1 Objectives.  To prove the non-inferiority of the fixed timolol-dorzolamide combination (Cosopt) compared to 0.005% latanoprost (Xalatan) in the aspects of intraocular pressure (IOP) and ocular perfusion pressure (OPP) in subjects with normal tension glaucoma (NTG)    2.2 Clinical hypotheses.  Primary hypothesis  : Cosopt group is non-inferior to Xalatan group in diurnal IOP reduction.    Secondary hypothesis  : Cosopt group is non-inferior to Xalatan group in diurnal diastolic and systolic OPP. |
| **2.2 Background & Rationale, Significance of Selected Topic & Preliminary Data** | Normal tension glaucoma (NTG) is a clinical entity characterized by an open angle and normal intraocular pressure (IOP), but resulting in progressive optic neuropathy. There are several factors that may be involved in the etiology of this optic neuropathy. Various publications suggest that NTG may be associated with vasospastic disorders,^1^ other studies emphasize the role of systemic blood pressure. Although several studies suggest other causes of NTG, IOP is the only treatable factor. In addition, CNTGS (Collaborative Normal Tension Glaucoma Study) showed progression of NTG could delay with IOP reduction.^2^  Cosopt (dorzolamide/timolol fixed combination) has well known effect for IOP reduction in POAG and OHT.^3-5^ However, there is no study about the effect and safety of Cosopt in NTG, Cosopt is not used the first line drug in the management of NTG.  In Korea, the prevalence of NTG is higher than western countries, the majority (about 80-90%) of open angle glaucoma patients have an IOP of 21 mmHg or less, so the study of NTG is important and searching of effective drug for the treatment of NTG is necessary.  In this study, we would prove the non-inferiority of Cosopt compared to Xalatan,^6^ which is used as a first line drug in the management of NTG, in the aspects of IOP and OPP including diastolic OPP (DOPP) |
| **2.3 Study Design** | A prospective, interventional, randomized, crossover, single masked, single center study. Forty-four NTG patients were randomly allocated to one of two groups. Patients in group A were treated with Cosopt, lubricant, and Xalatan for 4 weeks each, whereas patients in group B were treated with Xalatan, lubricant, and Cosopt for 4 weeks each.  2.3.1 Inclusion/ Exclusion criteria  The identification of NTG was based on reproducible glaucomatous visual field defects, corresponding to typical ONH changes.  Unilateral or bilateral visual field loss (description as below) as determined by at least two consecutive automated static threshold perimetry tests.  One eye is randomly selected in the cases where both eyes are treated.  Inclusion criteria were:  (1) age ranging from 45 to 75 years, (2) best-corrected visual acuity no worse than 20/30 Snellen equivalent, (3) optic nerve head cupping (i.e., a vertical cup-to-disc ratio of more than 0.6) and/or notching of neuroretinal rim, and/or retinal nerve fiber defects characteristic of glaucoma, (4) visual field loss (i.e., a localized defect with at least three adjacent nonedge points depressed >5 dB from the normal value, and a nucleus of at least one point depressed 10 dB from the normal value), (5) repeated measurements of untreated IOP, which documented values less than 22 mmHg, (6) central corneal thickness ranging from 540 to 560 microns, and (7) open-angle confirmed by gonioscopy.  Exclusion criteria were:  (1) active or chronic systemic diseases and/or concomitant assumption of any medication known to affect IOP, BP and/or HR, (2) corneal abnormalities preventing reliable applanation tonometry, (3) severe ocular trauma, ocular inflammation or infection, intraocular surgery or argon laser treatment or laser trabeculoplasty, (4) myopic or other fundus changes preventing reliable optic disc evaluation, (5) visual field defects caused by nonglaucomatous disease, and (6) history of allergy to the ingredients of Cosopt or Xalatan eye drops.  All the enrolled subjects gave their written informed consent to participate in the study, after a detailed description of the work objectives and of the procedures to be used. |
| **2.4 Study Flowchart** | **SUMMARY**  - Recruit newly diagnosed 44 NTG patients.  - Baseline diurnal IOP, systolic and diastolic BP will be measured.  - Forty-four NTG patients were randomly allocated to one of two groups.  - Patients in group A were treated with Cosopt, lubricant, and Xalatan for 4 weeks each, whereas patients in group B were treated with Xalatan, lubricant, and Cosopt for 4 weeks each.  - Patients were instructed to instill 1 drop of Xalatan at 8 pm.  - For Cosopt, patients were instructed to instill 1 drop at 8am and 8pm.  - The assessment schedule is presented in Table 1. Patients will be examined on day 1 ( without medication), week 4 ( under medication ), week 8 ( without medication), and week 12 ( under medication).  - On week 4 and week 12, diurnal IOP, systolic and diastolic BP, and OPP will be measured at 8am, 10am, noon, 4pm and 8pm.  - Statistical analysis between two groups.  4 weeks administration of Cosopt  Diurnal IOP and OPP check    Recruit 44 NTG patients  22 NTG patients in Group A  Baseline diurnal IOP and OPP check  22 NTG patients in Group B  4 weeks administration of Xalatan  Diurnal IOP and OPP check  Statistical analysis, adverse effect assessment  4 weeks of washout  Randomly allocated  : Group A  : Group B |
| **2.5 Study Procedures** | Table 1. -appendix  2.5.1. Recruit NTG patients  - Recruit newly diagnosis NTG patients, who are not treated with the glaucoma medication recent 2 months.  - Select the patients according to the inclusion and exclusion criteria as mentioned above  - Detailed explanation about the procedure and informed consent will be obtained.  - Baseline IOP, systolic and diastolic BP will be measured. (Day 1)  2.5.2. Measurement of IOP and OPP  - After 4 weeks of treatment of Cosopt or Xalatan, all participants will be checked diurnal IOP, systolic and diastolic BP.  - IOP was measured by Goldmann applanation tonometry (mean of three consecutive readings) with the patient in a sitting position at the slit lamp. Every IOP will be measured by one masked glaucoma specialist who is unaware of the treatment assignments.  - After the IOP measurements, after a 5-minite rest, pulse rate and BP (systolic and diastolic) of radial artery were measured in the sitting position using a standard automated blood pressure cuff. During the period, all measuring instruments keep to be calibrated by the manufacturer’s instruction.  - OPP was calculated according to the following formula:  OPP=(1/3 systolic BP + 2/3 diastolic BP) x 2/3 –IOP, diastolic OPP (DOPP)=diastolic BP-IOP.^6,7^  2.5.3. Washout period  - During the 4 weeks washout period, subjects will use lubricants.^8^(Week 8)  2.5.4. Alternative treatment  - In this crossover study, with the other eye drops, the same measurement will be performed. (Week 12)  2.5.5. Data analysis |
| **2.6 Study Duration** | To complete the study, about 13 months will be required.   - To recruit patients : 3 months - Treatment including washout period : 3 months - Data analysis and preparation of manuscript : 1 month - Submit for publication : 6months |
| **2.7 Statistical Analysis and Sample Size Justification** | - Analysis the study data will be performed by the investigator and department of biostatistics of the catholic university of Korea.^9^  Variables/Time Points of Interest  Differences among treatment regimens, clock hours, and their interaction will be assessed for the main outcome variables: IOP (mean IOP, peak IOP, trough IOP), diastolic and systolic BP, HR or OPP.  In all patients, baseline diurnal IOP, systolic and diastolic BP will be measured as baseline data. After 4 weeks administration of Cosopt or Xalatan, diurnal IOP, systolic and diastolic BP will be measured. After 4 weeks washout period, switching to the other eye drops, the same procedure will be performed.  Base on these data, OPP and DOPP will be calculated and statistical analysis of main outcomes will be performed.  Statistical Methods  To compare diurnal IOP and OPP of the two groups, repeated measured ANOVA will be used.  Power/Sample Size  To prove the non-inferiority of Cosopt to Xalatan, the sample size calculation was based on the assumption that non-inferiority margin of trough IOP of 1.5mmHg. A sample size of n=21 patients per group, this study has 80% power (1-β=0.80) and α=0.05, crossover-designed analysis.  In this study, the upper limit of the 95% CI is expected above the maximal acceptable clinically significant difference of 1.5 mmHg of IOP.  To calculate the sample size, we use the formula as below and review other crossover studies.^10,11^ Sample Size Calculator: Two Crossover-Sample Means **Application:** This procedure computes sample size for non-inferiority and superiority tests in 2×2 cross-over designs in which the outcome is a continuous normal random variable. A non-inferiority test tests that the treatment mean is not worse than the reference mean by more than a small equivalence margin. The actual direction of the hypothesis depends on the response variable being studied. A superiority test tests that the treatment mean is better than reference mean by more than a small equivalence margin. The actual direction of the hypothesis depends on the response variable being studied. The following hypotheses are usually considered: $H_{0}:\mu_{2}-\mu_{1}\leq\delta\text{ }versus\text{ }H_{a}:\mu_{2}-\mu_{1}>\delta$.  **Formula:**  **(*)**  **Notations:**  **α:** The probability of type I error (significance level) is the *probability of rejecting the true null hypothesis.*  **β:**   The probability of type II error (1 – power of the test) is the *probability of not rejecting the false null hypothesis.*  **δ:** The true difference between the two mean values at which the power is calculated.  **μ_2_ – μ_1_:** Margin of equivalence is the largest *change from the reference value (baseline) that is considered to be trivial.*  **n** : Sample size of each group |
| **2.8 Specific Drug Supply Requirements** | The drug supplies (Cosopt, Xalatan and lubricant) will be purchased locally as marketed products. |
| **2.9 Adverse Experience Reporting** | The study agreement outlines the requirement for adverse experience reporting. |
| **2.10 References** | 1. What Is the Present Pathogenetic Concept of Glaucomatous Optic Neuropathy? Surv Ophthalmol 2007;52:S162-S173. 2. Collaborative Normal Tension Glaucoma Study. Curr Opin Ophthalmol 2003;14:86-90. 3. Intraocular pressure lowering effect of dorzolamide/timolol fixed combination in patients with glaucoma who were unresponsive to prostaglandin analogs/prostamides. Curr Med Res Opin 2007;23:595-9  Comparison of the safety and efficacy of the fixed combination of dorzolamide/timolol and the concomitant administration of dorzolamide and timolol: a clinical equivalence study. International Clinical Equivalence Study Group. Br J Ophthalmol 1998;82:1249-53.  1. Topical dorzolamide 2%/timolol 0.5%: a review of its use in the treatment of open-angle glaucoma. Drugs Aging 2000;17:477-96. 2. Effect of latanoprost on the diurnal variations in the intraocular perfusion pressure in normal tension glaucoma. J Glaucoma 2006;15:354-7. 3. Effects of the Timolol-Dorzolamide Fixed Combination and Latanoprost on Circadian Diastolic Ocular Perfusion Pressure in Glaucoma. IOVS 2008;49:4226-31. 4. European Glaucoma Society. Antiglaucoma drugs. In :Terminology and Guidelines for Glaucoma. 2nd ed. Traverso CE, ed. European Glaucoma Society; 2003, Ch3-10-17. 5. On non-inferiority margin and statistical tests in active control trials. Statist Med 2006;25:1101-3. 6. Circadian changes of intraocular pressure and ocular perfusion pressure after timolol or latanoprost in Caucasians with normal tension glaucoma. Graefes Arch Clin Exp Ophthalmol 2008;246:389-96. 7. Changes in intraocular pressure and ocular perfusion pressure after latanoprost 0.005% or brimonidine tartrate 0.2% in normal tension glaucoma patients. Ophthalmol 2002;109:2241-7. |
| **2.11 Protocol Submission for Investigator-Initiated Studies** | Non U.S. protocols should be submitted to the MSD office by the investigators. |

|  | Day 1  (screening) | | | Week 4 | | | | | Week 8 | | | Week 12 | | | | |
| --- | --- | --- | --- | --- | --- | --- | --- | --- | --- | --- | --- | --- | --- | --- | --- | --- |
|  | 8  am | 12  pm | 4  pm | 8  am | 10  am | 12  pm | 4  pm | 8  pm | 8  am | 12  pm | 4  pm | 8  am | 10  am | 12  pm | 4  pm | 8  pm |
| Refraction | v |  |  | v |  |  |  |  | v |  |  | v |  |  |  |  |
| Visual acuity | v |  |  | v |  |  |  |  | v |  |  | v |  |  |  |  |
| Slit lamp exam | v |  |  | v |  |  |  |  | v |  |  | v |  |  |  |  |
| Fundus exam | v |  |  | v |  |  |  |  |  |  |  | v |  |  |  |  |
| History taking | v |  |  | v |  |  |  |  |  |  |  | v |  |  |  |  |
| Symptoms | v |  |  | v | v | v | v | V | v |  |  | v | v | v | v | v |
| IOP check | v | v | v | v | v | v | v | v | V | v | v | v | v | v | v | v |
| Pulse rate | v | v | v | v | v | v | v | v | V | v | v | v | v | v | v | v |
| SBP & DBP | v | v | v | v | v | v | v | V | v | v | v | v | v | v | v | v |
| **Drug Supply** |  |  |  | Cosopt for Group A,  Xalatan for Group B | | | | | Lubricant | | | Xalatan for Group A,  Cosopt for Group B | | | | |

Table 1. Check list for normal tension glaucoma patients with Cosopt or Xalatan treatment
