# Supplementary material for: Comparison of the Effects of Dorzolamide/Timolol Fixed Combination versus Latanoprost on Intraocular Pressure and Ocular Perfusion Pressure in Patients with Normal-Tension Glaucoma: A Randomized, Crossover Clinical Trial
Source: PLoS One. 2016 Jan 12;11(1):e0146680. doi: 10.1371/journal.pone.0146680 (PMC4710520; doi:10.1371/journal.pone.0146680)
Supplement: S2 Text — (DOC) [file pone.0146680.s002.doc]

| **임상연구 계획서** |
| --- |
| (1)임상연구의 명칭 및 단계 |
| 정상안압녹내장 환자에서 잘라탄과 비교한 코솝 안약 점안시의 안압 및 안관류압 변화 및 효과에 대한 연구 |
| (2)임상연구 실시기관명 및 주소 |
| 서울성모병원, 서울시 서초구 반포동 505 서울성모병원 |
| (3)연구의 책임자 및 담당자/공동연구자 성명, 직위, 소속 |
| 박찬기/교수/서울성모병원  이나영/전임강사/인천성모병원  박혜영/임상강사/서울성모병원 |
| (4)의뢰자(기관)명 및 주소 |
| Merck Sharp&Dohme (MSD), Whitehouse station, N.J., USA |
| (5) 임상연구의 목적 |
| 정상안압녹내장 환자에서 코솝 (timolol/dorzolamide 복합제제) 점안에 의한 안압 및 안관류압 변화를 잘라탄 (0.005% latanoprost) 점안과 비교하여 알아보고자 한다. |
| (6) 임상연구의 배경 |
| 정상안압녹내장은 정상 안압에도 불구하고 진행성의 시신경병증을 보이는 질환이다. 이러한 시신경병증에 관여하는 요인은 여러 가지가 제시되고 있다. 그 중에서도 혈관연축성 질환과의 연관성이 많이 발표되고 있으며, 전신혈압과의 중요성 또한 강조되고 있다. 하지만 이러한 여러 위험인자들에도 불구하고 안압이 가장 중요하고, 또 유일한 치료 가능한 인자로 생각된다. 정상안압녹내장 환자를 대상으로 한 대규모 연구였던 CNTGS (Collaborative Normal Tension Glaucoma Study)에서도 안압 하강이 정상안압녹내장의 진행을 늦추는 것으로 발표되었다.  코솝은 원발성 개방각 녹내장이나 고안압증 환자에서 효과적인 안압 하강을 보인 약물이다. 하지만 아직 정상안압녹내장 환자에서의 안압 하강 효과나 안정성에 대한 연구가 없으며, 아직까지 정상안압녹내장에서는 첫번째 시도되는 약물로 정립되지 않았다. 우리나라는 정상안압녹내장의 유병율이 서양에 비해 높으며, 개방각 녹내장의 80-90%가 안압이 21 mmHg 이하이다. 따라서 정상안압녹내장에 대한 연구가 중요하고 이들의 치료에 효과적인 약물의 발견이 중요하다고 할 수 있다. 본 연구에서는 현재 정상안압녹내장의 첫번째 치료 약물인 잘라탄과 비교한 코솝의 치료 효과를 안압 및 안관류압 (특히 이완기 안관류압을 포함한) 면에서 보고자 한다. |
| (7) 연구 대상 |
| 최근 2개월 간 녹내장 치료를 받은 적이 없는 정상안압녹내장 환자들  (만약 녹내장 치료를 받은 적이 있다면 1달 간의 wash out 기간을 거친 후 연구에 포함) |
| (8) 피험자의 선정기준, 제외기준, 목표한 피험자의 수 및 그 근거 |
| 피험자 선정기준 :   - 정상안압녹내장에 합당한 시신경병증 및 시야결손이 있는 환자 - 단안 또는 양안의 녹내장성 시야결손이 최소한 두 번의 연속적인 시야검사 상 나타나는 환자 - 양안이 정상안압녹내장일 경우, 단안만 무작위로 연구에 포함시킴   이 조건을 충족시키는 환자 중 다음의 기준에 맞는 환자가 연구에 포함된다 : (1) 나이가 45-75세, (2) 최대정시력 20/30 Snellen equivalent 이상, (3) 시신경 유두 함몰비가 0.6 이상이면서 시신경 유두테의 국소적인 결손이나 패임이 있는 경우 (4) 시야결손에서 활모양영역에 있는 3개 이상의 점의 역치가 정상의 5% 미만에서 나타나고 그 중 한 점이 1% 미만일 때, (5) 반복적인 안압 측정에서 안압이 22 mmHg 미만일 때, (6) 중심각막두께가 540-560 microns, (7) 전방각경 검사상 개방각일 때.  제외기준은 :  (1) 급성 또는 만성질환이 있으면서 안압이나 혈압에 영향을 줄 것으로 추정되는 약물을 사용중인 경우, (2) 안압 측정의 오류를 가져올 수 있는 각막 이상이 있는 경우, (3) 안 외상이나 염증, 감염, 안내 수술 또는 레이저의 과거력이 있는 경우, (4) 정확한 시신경 검사를 저해하는 근시나 망막의 변화가 있는 경우, (5) 녹내장 이외의 원인에 의한 시야결손이 있는 경우, (6) 코솝이나 잘라탄에 대한 부작용이 있었던 경우.  목표한 피험자의 수 및 산출 근거  목표한 피험자의 수는 44명이며, 중간 탈락환자를 고려하여 50명을 연구에 포함시킬 예정이다. 두 약물을 사용한 군 간의 차이를 보기 위한 최소한의 안압 효과 차이를 1.5 mmHg로 두고 산출하였다. 각 군의 피험자 수가 21명일 경우, 이 연구는 80% power (1-β=0.80) 및 α=0.05의 crossover-designed 연구이다. 또한 이 연구에서 95% CI의 상부한계는 안압의 1.5 mmHg 차이를 볼 수 있는 수준으로 정하였다. 목표 피험자 수를 산출하기 위해 아래의 식을 사용하였으며 다른 crossover 연구들을 고찰하였다. Sample Size Calculator: Two Crossover-Sample Means **Application:** This procedure computes sample size for non-inferiority and superiority tests in 2×2 cross-over designs in which the outcome is a continuous normal random variable. A non-inferiority test tests that the treatment mean is not worse than the reference mean by more than a small equivalence margin. The actual direction of the hypothesis depends on the response variable being studied. A superiority test tests that the treatment mean is better than reference mean by more than a small equivalence margin. The actual direction of the hypothesis depends on the response variable being studied. The following hypotheses are usually considered:  $H_{0}:\mu_{2}-\mu_{1}\leq\delta\text{ }versus\text{ }H_{a}:\mu_{2}-\mu_{1}>\delta$.  **Formula:**  **(*)**  **Notations:**  **α:** The probability of type I error (significance level) is the *probability of rejecting the true null hypothesis.*  **β:**   The probability of type II error (1 – power of the test) is the *probability of not rejecting the false null hypothesis.*  **δ:** The true difference between the two mean values at which the power is calculated.  **μ_2_ – μ_1_:** Margin of equivalence is the largest *change from the reference value (baseline) that is considered to be trivial.*  **n** : Sample size of each group |
| (9) 임상연구의 기간 |
| 임상연구기간은 약 8개월   - 피험자 모집 : 4개월 - 약물 투여 및 관찰 기간 : 3개월 - 자료 분석 및 준비 : 1개월 |
| (10)임상연구의 방법 |
| 50명의 정상안압녹내장 환자를 연구에 포함시킨 후 무작위로 두 군으로 나누어 배정한다. 군의 분배는 무작위로 1:1로 나누어 배정한다. A군은 코솝 안약-라큐아 눈물 안약-잘라탄 안약 순으로, B군은 잘라탄 안약-라큐아 눈물 안약-코솝 안약 순으로 치료를 한다.  1. 피험자 수집  - 정상안압녹내장으로 진단받은 환자 중에서 선정기준을 충족하는 환자는 모두 대상이 됨  - 연구 수행과정의 상세한 설명 후 동의를 구하고 본인 의사에 의해 연구에 참여할 뜻을 보인 환자에 한하여 동의서를 받고 연구에 포함시킴.  - 안압, 수축기 및 이완기 혈압을 측정함 (Day 1)  2. 안압 및 안관류압 측정  - 연구에 포함된 날부터 코솝 또는 잘라탄 점안 (안약의 점안은 환자가 직접하며, 코솝은 하루 12시간 간격으로 2번 점안, 잘라탄은 하루 1번 자기 전에 점안함)을 하고, 4주 후 내원하여 안압, 수축기 및 이완기 혈압을 하루 5회 측정함 (Week 4).  - 안압은 앉은 자세에서 골드만 압평 안압계로 세 번 측정한 평균 안압을 구한다.  - 안압 측정 후 5분 안정 후 자동혈압계로 혈압을 구한다.  - 안관류압은 다음과 같은 식에 의해 계산한다 :  OPP=(1/3 systolic BP + 2/3 diastolic BP) x 2/3 –IOP, diastolic OPP (DOPP)=diastolic BP-IOP  3. Washout period  - 4 주 동안의 washout period를 가지며 라큐아 눈물 안약을 사용한다 (Week 8).  4. 치료의 교체  - 코솝 사용 군은 잘라탄으로 (A군), 잘라탄 사용 군을 코솝으로(B군) 교체 치료한다 (Week 12)  4 weeks administration of Cosopt  Diurnal IOP and OPP check    Recruit 44 NTG patients  22 NTG patients in Group A  Baseline diurnal IOP and OPP check  22 NTG patients in Group B  4 weeks administration of Xalatan  Diurnal IOP and OPP check  Statistical analysis, adverse effect assessment  4 weeks of washout  Randomly allocated  : Group A  : Group B |
| (11) 관찰항목, 임상검사항목 및 관찰 검사 방법 |
| |  | Day 1  (screening) | | | Week 4 | | | | | Week 8 | | | Week 12 | | | | | | --- | --- | --- | --- | --- | --- | --- | --- | --- | --- | --- | --- | --- | --- | --- | --- | --- | |  | 8  am | 12  pm | 4  pm | 8  am | 10  am | 12  pm | 4  pm | 8  pm | 8  am | 12  pm | 4  pm | 8  am | 10  am | 12  pm | 4  pm | 8  pm | | *굴절검사* | v |  |  | v |  |  |  |  | v |  |  | v |  |  |  |  | | *시력검사* | v |  |  | v |  |  |  |  | v |  |  | v |  |  |  |  | | *세극등 현미경 검사* | v |  |  | v |  |  |  |  | v |  |  | v |  |  |  |  | | *안저 검사* | v |  |  | v |  |  |  |  |  |  |  | v |  |  |  |  | | *문진* | v |  |  | v |  |  |  |  |  |  |  | v |  |  |  |  | | *증상 질문* | v |  |  | v | v | v | v | V | v |  |  | v | v | v | v | v | | *안압 측정* | v | v | v | v | v | v | v | v | V | v | v | v | v | v | v | v | | *맥박 측정* | v | v | v | v | v | v | v | v | V | v | v | v | v | v | v | v | | *현압 측정* | v | v | v | v | v | v | v | V | v | v | v | v | v | v | v | v | | ***안약 점안*** |  |  |  | Cosopt for Group A,  Xalatan for Group B | | | | | Lubricant (Lacure) | | | Xalatan for Group A,  Cosopt for Group B | | | | | |
| (12) 중지 및 탈락 기준 |
| - 피험자가 원하는 경우 - 안약 부작용으로 계속 사용하기 어려운 경우 - 피험자가 내원일에 오지 못하여 필요한 검사를 시행하지 못한 경우 |
| (13) 효과 평가기준, 평가방법, 해석방법(통계분석방법) |
| 통계적인 분석은 가톨릭대학교 통계학 교실에 의뢰하여 분석할 예정  평가 기준  두 약물 사용 군간의 안압 (평균, 최고, 최저 안압), 수축기 및 이완기 혈압, 안관류압을 분석한다. 주된 비교 항목은 안관류압 및 이완기 안관류압이 된다.  통계분석방법  두 군간의 평가항목을 repeated measured ANOVA로 분석한다. |
| (15) 피험자의 안전보호에 대한 대책 (별표 4) 연구자의 서약서 첨부 |
| 안약의 부작용이 발생할 경우 즉각적인 조치를 취함 |
| (16) 증례기록서 양식(별첨할 것) |
| 첨부함 |
| (18) 해당연구의 근거가 되는 임상문헌(참고 문헌) |
| 1. What Is the Present Pathogenetic Concept of Glaucomatous Optic Neuropathy? Surv Ophthalmol 2007;52:S162-S173. 2. Collaborative Normal Tension Glaucoma Study. Curr Opin Ophthalmol 2003;14:86-90. 3. Intraocular pressure lowering effect of dorzolamide/timolol fixed combination in patients with glaucoma who were unresponsive to prostaglandin analogs/prostamides. Curr Med Res Opin 2007;23:595-9  Comparison of the safety and efficacy of the fixed combination of dorzolamide/timolol and the concomitant administration of dorzolamide and timolol: a clinical equivalence study. International Clinical Equivalence Study Group. Br J Ophthalmol 1998;82:1249-53.  1. Topical dorzolamide 2%/timolol 0.5%: a review of its use in the treatment of open-angle glaucoma. Drugs Aging 2000;17:477-96. 2. Effect of latanoprost on the diurnal variations in the intraocular perfusion pressure in normal tension glaucoma. J Glaucoma 2006;15:354-7. 3. Effects of the Timolol-Dorzolamide Fixed Combination and Latanoprost on Circadian Diastolic Ocular Perfusion Pressure in Glaucoma. IOVS 2008;49:4226-31. 4. European Glaucoma Society. Antiglaucoma drugs. In :Terminology and Guidelines for Glaucoma. 2nd ed. Traverso CE, ed. European Glaucoma Society; 2003, Ch3-10-17. 5. On non-inferiority margin and statistical tests in active control trials. Statist Med 2006;25:1101-3. 6. Circadian changes of intraocular pressure and ocular perfusion pressure after timolol or latanoprost in Caucasians with normal tension glaucoma. Graefes Arch Clin Exp Ophthalmol 2008;246:389-96.   11. Changes in intraocular pressure and ocular perfusion pressure after latanoprost 0.005% or brimonidine tartrate 0.2% in normal tension glaucoma patients. Ophthalmol 2002;109:2241-7. |

- 해당사항이 없는 항목은 ‘해당사항 없음’으로 기재해 주십시오.
- 임상시험/연구와 관련하여 보충 설명/자료가 필요한 경우 별첨하여 주시기 바랍니다.
